# Outcome of prosthesis exchange for infected knee arthroplasty: the effect of treatment approach: A systematic review of the literature

**DOI:** 10.1080/17453670902805064

**Published:** 2009-02-01

**Authors:** Esa Jämsen, Ioannis Stogiannidis, Antti Malmivaara, Jorma Pajamäki, Timo Puolakka, Yrjö T Konttinen

**Affiliations:** ^1^Medical School, University of TampereTampereFinland; ^2^Coxa, Hospital for Joint ReplacementTampereFinland; ^3^The Finnish Office for Health Technology AssessmentHelsinkiFinland; ^4^Department of Medicine, Helsinki University Central HospitalHelsinkiFinland

## Abstract

**Background and purpose** Two-stage revision remains the gold standard in the treatment of infected knee arthroplasty. Lately, good long-term results of direct exchange arthroplasty have been reported. The purpose of this literature review is to compare the clinical outcome achieved with one-stage revision and two-stage revision with different types of spacers.

**Methods** A thorough systematic review of literature was undertaken to idenepsy reports on the treatment alternatives. Papers written in English or including an English abstract, published from 1980 through 2005, and reporting either the success rate in eradication of infection or the clinical status achieved were reviewed. 31 original articles describing the results of 154 one-stage exchange arthoplasties and of 926 two-stage exchange arthoplasties were included. The depth of detail in the description of materials and methods varied markedly, making it impossible to perform a meta-analysis. Instead, a descriptive review of the results is presented.

**Results** With a follow-up of 12–122 months, the overall success rate in eradication of infection was 73–100% after one-stage revisions and 82–100% after two-stage revisions. Reinfection rates were the lowest in series where articulating cement spacers were used, though the follow-up was relatively short. Studies using articulating spacers reported the highest average postoperative ranges of motion. Otherwise, no correlations were observed between the clinical outcome and the length of follow-up, the type of revision, or the type of spacer. The clinical outcome (knee scores and range of motion) of the one-stage revisions was no different from that of the two-stage revisions.

**Interpretation** Two-stage exchange is an effective treatment. Mobile spacers may further improve the range of motion. More experience in one-stage revision is required in order to define its role in the management of infected knee arthroplasties.

## Introduction

Two-stage exchange arthroplasty has remained the gold standard in the treatment of infected total knee arthroplasties (TKAs) for over two decades (Leone and Hanssen 2006). The original concept (Insall et al. 1982, 1983) has been modified by several authors. Spacer block technique was first developed to avoid scarring of the joint during the interim period, and to work as a local antibiotic carrier (Borden and Gearen 1987, Cohen et al. 1988). Later, articulating spacers were introduced to enhance the patient’s functional status and to maintain the range of motion during the period between the two stages (Masri et al. 1994, Hofmann et al. 1995, Fehring et al. 2000) to improve the often poor knee score and patient satisfaction (Barrack et al. 2000b, Wang et al. 2004). Irrespective of the type of spacer used, satisfactory outcomes in terms of infection eradication have been reported.

Very little has been published on one-stage revision in the literature (Silva et al. 2002), but promising long-term results have been reported (Buechel et al. 2004). The studies that have been published suggest that direct exchange may have a role in the management of infected knee arthroplasties, but this role is not clear at present. It is also unclear whether the type of spacer used in two-stage revision affects the outcome. Thus, we performed this systematic review to assess the effect of treatment approach on the outcome of infected knee arthroplasty.

## Material and methods

In January 2006, we searched several international databases using highly sensitive though somewhat unspecific search strategies, which are described in detail in Appendix 1. The literature search strategies were created in co-operation with a librarian. The research plan was reviewed and approved by the Finnish Office for Health Technology Assessment (see http://finohta.stakes.fi/).

Two of the present authors (EJ and IS) reviewed the search results independently and classified the references found in terms of whether they should be included on basis of the title of the paper (Table 1), whether they should be excluded, or whether this was unclear. Abstracts of unclear references were reviewed and papers were then classified as being included or excluded. In addition to original study reports, review articles and articles dealing with the treatment costs of infected knee replacements were reviewed. Reference lists from all reviewed articles were assessed to complete the literature search. The two reviewers’ lists of papers that should be included were compared to each other, and where there was any discrepancy, they were re-classified according to the consensus reached. The list of articles included was reviewed by an expert on the subject who did not belong to the study group and it was found to fulfill the inclusion criteria.

**Table 1.** The inclusion criteria for original papers

The report concerns the results of management of infected knee arthroplasty only with one-stage or two-stage exchange arthroplasty.
The study can be classified into one of the following groups:
– randomized controlled trial
– prospective study comparing two simultaneous treatment groups
– prospective study with historical controls
– prospective case series with no comparison group
– retrospective study comparing two simultaneous treatment groups
– retrospective study with historical control group
– retrospective study with no control group
Study includes more than 5 cases treated.
The paper is written in English or it has an abstract in English.
One or more of the following outcome variables is reported:
– number of all infections appearing after the treatment
– number of reinfections
– clinical status at follow-up, reported using Hospital for Special Surgery or Knee Society knee score
– range of motion at follow-up

Finally, 25 original studies published in English (Rosenberg et al. 1988, Booth and Lotke 1989, Henderson et al. 1991, Göksan and Freeman 1992, Masri et al. 1994, Whiteside 1994, Gusso et al. 1995, Goldman et al. 1996, McPherson et al. 1997, Hirakawa et al. 1998, Fehring et al. 2000, Haddad et al. 2000, Mont et al. 2000, Lonner et al. 2001, Emerson et al. 2002, Siebel et al. 2002, Jhao and Jiang 2003, Buechel et al. 2004, Durbhakula et al. 2004, Haleem et al. 2004, Meek et al. 2004, Cuckler 2005, Hofmann et al. 2005, MacAvoy and Ries 2005, Pitto et al. 2005) and 6 studies published in other languages (von Foerster et al. 1991, Gacon et al. 1997, Lu et al. 1997, Lecuire et al. 1999, Kirschner et al. 2000, Pietsch et al. 2003) between 1983 and 2005 were included. The materials of several studies (Insall et al. 1983, Wilde and Ruth 1988, Hofmann et al. 1995, 2005, Goldman et al. 1996, Hirakawa et al. 1998, Windsor et al. 2000, Meek et al. 2003, 2004) were identical. In such cases, the most recent report (Goldman et al. 1996, Hirakawa et al. 1998, Meek et al. 2004) was accepted for further analysis. In addition, reports by Booth and Lotke (1989) and Henderson and Booth (1991) presented partly overlapping data, but these studies were reviewed separately.

Data about materials, methods, and results of each original article included was extracted into a specific form independently by EJ and IS. Each study was assessed using a checklist (Appendix 2). The checklist, based on previously published criteria for evaluation of the internal validity (van Tulder et al. 1997) and generalizability (Malmivaara et al. 2006) of studies in systematic reviews, was customized for the context of this review. The quality score calculated was not used as an exclusion criterion.

The materials extracted by EJ and IS were compared to each other and conflicting data were re-checked from the original papers and corrected after discussion.

The principal outcomes were (1) the rates of new and recurrent infections and (2) the clinical outcome—measured as postoperative clinical knee score and range of motion—following the revision arthroplasty. Due to heterogeneity and to the low calculated quality scores in the reports, the study materials were not pooled using meta-analytical techniques. Instead, several graphs were prepared for the principal outcome variables in order to visualize any possible trend after sorting the materials by a potentially explanatory variable. A qualitative and descriptive summary of the results is also presented.

## Results

The studies reported the results of 926 two-stage and 152 one-stage arthroplasties (Table 2). The number of operations reported in any one study varied between 5 and 104, and the average length of follow-up ranged from 12 to 122 months. In total, 3,718 and 986 case-years of follow-up were reported for two-stage and one-stage revisions, respectively.

**Table 2.** Original studies included: materials and outcome

Study				Materials					Outcome	
		A	B	C	D	E	F	G	H	I
Rosenberg et al.	1988	26	1981–1986	65/31/4	67	2-stage, no spacer	29 (12–57)	100	78	NA
Booth and Lotke ^a^	1989	25	1984–1988	NA	67	2-stage, PMMA block	25 (6–59)	96	82	100
Henderson and Booth ^a^	1991	28	1984–1989	89/11/0	73	2-stage, PMMA block	27 (12–79)	97	86	90
von Foersteret et al. ^c^	1991	104	1976–1985	61/29/10	NA	1-stage	76 (60–180)	73	NA	NA
Göksan and Freeman	1992	18	1979–1989	39/56/5	61	1-stage	60 (12–120)	94	NA	85
Masri et al.	1994	24	1987–1993	75/8/17	66	2-stage, PROSTALAC	26 (6–73)	96	80	86
Whiteside	1994	33	NA	85/15/0	NA	2-stage, PMMA block	24	97	NA	98
Gusso et al.	1995	5	NA	80/NA/NA	NA	2-stage, PMMA block	(4–18)	100	78	105
Goldman et al.	1996	64	1977–1993	70/25/5	67	2-stage, no spacer ^b^	90 (24–204)	97	78	94
Gacon et al. ^c^	1997	29	1984–1994	97/3/0	70	2-stage, PMMA block	42	83	80	95
Lu et al. ^c^	1997	8	NA	NA	NA	1-stage	20	100	NA	NA
McPherson et al.	1997	21	1993–1996	NA	64	2-stage, no spacer	17 (5–33)	95	77	99
Hirakawa et al.	1998	55	1980–1993	75/25/0	67	2-stage, PMMA block	NA	82	79	83
Lecuire et al. ^c^	1999	12	1989–1998	NA	NA	2-stage, no spacer	12	100	NA	96
Fehring et al.	2000	25	1986–1995	NA	68	2-stage, PMMA block	36 (24–72)	88	83	98
		15	1996–1999	NA	NA	2-stage, artic. PMMA	27 (24–36)	93	84	105
Haddad et al.	2000	45	1987–1996	80/13/7	69	2-stage, PROSTALAC	48 (20–112)	98	72	95
Kirschner et al. ^c^	2000	6	1996–1997	NA	62	2-stage, artic. PMMA	19 (13–21)	100	NA	NA
Mont et al.	2000	69	1989–1993	NA	66	2-stage, PMMA block	63 (36–114)	93	86	96
Lonner et al.	2001	53	1983–1997	NA	NA	2-stage, PMMA block	56 (24–144)	91	NA	NA
Emerson et al.	2002	26	1986–1994	NA	66	2-stage, PMMA block	90 (34–152)	92	NA	94
		22	1995–1999	NA	65	2-stage, RPS	46 (31–77)	100	NA	108
Siebel et al.	2002	10	NA	NA	66	2-stage, artic. PMMA	18 (6–26)	100	64	87
Jhao and Jiang	2003	7	1994–2001	86/14/0	68	2-stage, no spacer	42 (12–84)	100	86	91
Pietsch et al. ^c^	2003	24	1999–2002	NA	d	2-stage, RPS	15 (5–33)	96	NA	NA
Haleem et al.	2004	96	1989–1994	77/15/8	69	2-stage, PMMA block	86 (30–158)	96	50	90
Buechel et al.	2004	22	1981–1993	95/5/0	71	1-stage	122 (17–235)	100	80	NA
Durbhakula et al.	2004	24	1998–2001	NA	72	2-stage, artic. PMMA	33 (28–51)	100	82	104
Meek et al.	2004	54	1997–1999	NA	NA	2-stage, PROSTALAC	41	96	76	87
Cuckler	2005	44	1994–2002	NA	68	2-stage, RPS	65 (24–120)	100	84	112
Hofmann et al.	2005	50	1989–2001	NA	67	2-stage, RPS	74 (24–150)	94	89	4–104
MacAvoy and Ries	2005	13	NA	NA	58	2-stage, B&S	28 (15–44)	100	NA	98
Pitto et al.	2005	21	2000–2003	NA	67	2-stage, artic. PMMA	24 (12–43)	100	81	94

OA: osteoarthritis; RA: rheumatoid arthritis; ROM: range of motion; NA: not available; PMMA: polymethylmethacrylate bone cement; PROSTALAC: prosthesis of antibiotic-loaded cement; RPS: resterilized prosthesis spacer; B&S: ball-and-socket spacer;

^a^ partly overlapping data;

^b^ includes 7 knees with PMMA block,

^c^ non-English publications.

^d^ range 42–78

A No. of knees

B Year(s) of collection

C Diagnosis OA/RA/other, %

D Average age at operation, years

E Revision type, spacer

F Length of follow-up, months (range)

G Rate of eradication of infection, %

H Average clinical knee score postoperatively

I ROM or flexion postoperatively, degrees

### Study methodology and quality

None of the studies included used a randomized or controlled trial setting. In 2 studies of two-stage revisions, historical comparison groups were used to analyze the effect of spacer type on treatment outcome (Fehring et al. 2000, Emerson et al. 2002). 5 studies stated that the materials had been collected in a prospective manner (Whiteside 1994, Goldman et al. 1996, Mont et al. 2000, Siebel et al. 2002, Pitto et al. 2005). In a considerable number of studies, either materials or the treatment intervention—or both—was incompletely described (Table 3). All the outcome variables of interest for this systematic review were reported in only 10 series. The total quality score ranged from 8 to 17 out of a maximum of 20 points. Quality score was not calculated for the 6 reports in languages other than English.

**Table 3.** Quality scores of the 25 studies published in English that were included. Values are number of studies

Quality measure	Measure reported	Measure not reported
Materials		
Average age	21	4
Male-to-female ratio	23	2
Primary diagnoses	13	12
ASA or other risk classification	2	23
Pathogens	23	2
Preoperative clinical knee score	9	16
Preoperative range of motion or flexion range	9	16
Intervention		
Peroperative antibiotic treatment	19	6
Type of spacer	22	1
Mean time between the two stages	16	7
Postoperative antibiotic treatment	9	16
Rehabilitation	6	19
Results		
No. of all new infections	25	0
No. of reinfections/recurrent infections	24	1
No. of other failures	11	14
Postoperative clinical knee score	20	5
Postoperative range of motion/flexion	23	2
	True	False
Methodology		
Were both advantages and disadvantages of the treatment described?	8	17
Was the number of patients excluded less than 20% of the number of patients included?	21	4
Was the species of pathogen idenepsied used as an exclusion criterion?	3	22
Was loss to follow-up less than 20%?	25	0

### Treatment interventions

Table 2 gives a summary of materials and treatment approaches of the studies reviewed. Measured by the number of cases and the total case-years of follow-up, best experience has been reported with static polymethylmethacrylate (PMMA) spacers (52 to 691 case-years of follow-up). Medium- to long-term follow-up has also been reported for resterilized prosthesis spacers (RPSs, resterilized removed femoral components with either a new or resterilized tibial polyethylene insert) and one-stage revision. The experience is shortest with articulating PMMA spacers (10–66 case-years) and with the ball-and-socket spacer, used to manage knees with remarkable ligamentous laxity and bony defects (30 case-years).

In all studies, patients were given intravenous antibiotics after the first-stage operation, usually for 4–6 weeks. The details of antibiotic treatment (average length, antibiotic selection, use of oral antibiotics) were often unavailable, however, and only half of the studies reported the exact length of the interim period between the two stages (range of averages, 43–175 days). After reimplantation arthroplasty, a common protocol seems to have been to continue intravenous antibiotics until the results of operative bacterial cultures were ready. There appeared to be a correlation between publication year (rather than spacer type) and the duration of the interim period, with the most recent studies reporting shortest interim periods (data not shown). After one-stage revisions, antibiotics were given intravenously for periods ranging from 1–2 weeks (Göksan and Freeman 1992) to 4–6 weeks (Buechel et al. 2004).

There were no statistically significant differences in patient demographics (Table 2), types of detected pathogens (the proportion of staphylococcal species was 48–85%), or the quality score of the studies (not shown) between the studies concerning different spacer types or between the studies on two-stage or direct revision. The type of infection (acute vs. chronic) and the time from index arthroplasty to resection of the infected prosthesis were reported too seldom to enable comparison of the studies in these respects.

### Eradication of infection

**Figure 1.**
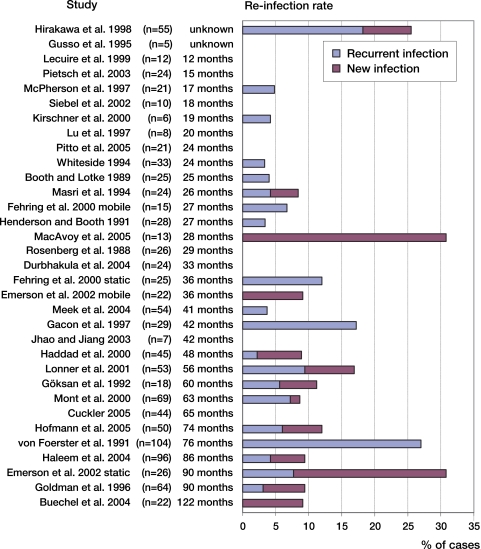
Rates of recurrent and new infections. Studies sorted by length of follow-up.

Success in infection management was analyzed in three ways. Firstly, the total number of infections appearing after treatment was recorded, supplemented by the number of recurrent infections and of new infections (that is, postoperative infections caused by an organism other than the one detected upon treatment). After the index revision, deep knee infection was detected in 0–31% of cases. The treated infections recurred in 0–18% of the cases. The new infection rate varied from 0 to 31%.The length of follow-up did not appear to affect the rate of recurrent infections, but the reports with follow-up of < 4 years had few new infections (Figure 1).

**Figure 2.**
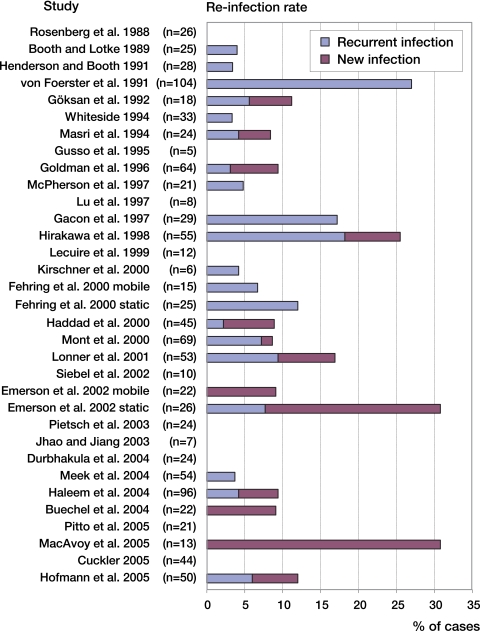
Rates of recurrent and new infections after revision arthroplasty for infection. Series sorted by publication year.

The lowest rates of recurrent infection were reported in studies where no spacer was used (0–5%) (Table 2). Also, resterilized prosthesis spacers, articulating PMMA spacers, and one-stage revision groups had low recurrence rates (0–6%, 0–7%, and 0–6%, respectively). No association was seen between type of spacer and the rate of new infections, although in the ball-and-socket spacer series with relatively young patients an exceptionally high number of new infections was reported (31%). The indication for primary arthroplasty (osteoarthritis or other), mean age, or the pathogen idenepsied did not appear to affect the incidence of post-revision infection rates (data not shown). When the series were sorted by the year of publication, a decline in recurrent infection rate was seen in the most recent studies (Figure 2).

### Clinical outcome

Knee Society knee score and Hospital for Special Surgery knee score were used to measure the clinical outcome in most studies. Even more frequently, postoperative range of motion or maximal flexion was reported (Table 3). Preoperative scores were reported in 9 studies only. Comparisons between different treatment approaches were made in two ways: firstly, the studies were grouped by treatment approach (one vs. two stages) and spacer ideology (no spacer, static spacer, articulating spacer), and then different spacer types were analyzed separately.

**Figure 3.**
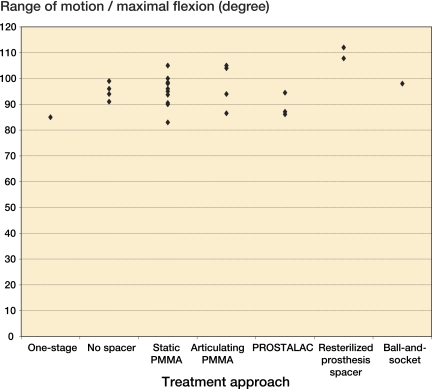
The effect of treatment approach on the average postoperative range of motion or maximal flexion. Each dot represents one study.

The highest average postoperative ranges of motion or maximal flexion exceeding 100 degrees (104–112 degrees) were achieved with articulating spacers (Fehring et al. 2000, Emerson et al. 2002, Durbhakula et al. 2004, Cuckler 2005), but the small study by Gusso et al. (1995) also reported average flexion of 105 degrees (Figure 3). Other studies reported values ranging from 80 to 100 degrees. Despite the type of spacer, there was considerable variance so no definitive conclusions can be drawn. Most studies reported an average clinical score of at least 80 out of 100. No obvious trend was seen when clinical outcome was compared to any of the various explanatory variables (length of follow-up, revision type, spacer type). There was no difference in outcome with one-stage revisions and two-stage revisions.

Data that can allow calculation of the change from preoperative to latest postoperative range of motion or clinical score were available in 9 studies. Except for 1 report (Hirakawa et al. 1998), considerable improvement was seen irrespective of the spacer type. The highest improvements in clinical scores were achieved with static PMMA spacers (Gusso et al. 1995, Haleem et al. 2004) or resterilized prosthesis spacers (Cuckler 2005), while the change in range of motion was highest in series with articulating spacers (Haddad et al. 2000, Emerson et al. 2002, Pitto et al. 2005). Hirakawa et al. (1998) reported a series of 55 cases with static PMMA spacers, with an average follow-up of 62 months. During the follow-up, a decline in both clinical score (–6.7 points) and range of motion (–9 degrees) was seen.

2 studies comparing static and mobile spacers have been published (Fehring et al. 2000, Emerson et al. 2002). Clinical scores were not reported in these studies. In both studies, the static spacer group comprised a number of historical controls treated with static PMMA spacer. There was no statistically significant difference between shaped articulating PMMA spacers and static spacers regarding postoperative range of motion (Fehring et al. 2000). In contrast, articulating spacers made of resterilized prosthesis components resulted in superior postoperative range of motion (Emerson et al. 2002). This led the authors to conclude that the more the spacer resembles a real total knee prosthesis, the better the clinical outcome will be. In both studies the use of a historical control group introduces a time period effect as a confounding factor, which may partly explain the difference.

## Discussion

Despite the relatively high number of reports on the treatment of infected TKAs with exchange arthroplasty, there is a lack of prospective comparative trials. Most of the studies reviewed were retrospective case series and failed to describe materials, surgical technique, or both in enough detail—thus making it difficult to compare the studies to each other. The studies published after the literature searches of our study (Hart and Jones 2006, Huang et al. 2006, Jämsen et al. 2006, Pietsch et al. 2006, Souillac et al. 2006, Trezies et al. 2006, Hsu et al. 2007, Mittal et al. 2007) do not appear to be any better in this respect (Table 4).

**Table 4.** Overview of studies published after the literature search

A		B	C	D	E	F	G	H	I
Hart and Jones	2006	48	1998–2003	articulating PMMA	49 (26–85)	88	NA		c
Huang et al.	2006	21	1996–2002	RPS	52 (30–102)	95	85	81 ^a^	
Jämsen et al.	2006	34	1993–2003	RPS (n = 24)	25 (2–68)	91	104	82 ^a^	
				static PMMA (n = 10)	49 (2–86)	75	92	79 ^a^	
Trezies et al.	2006	11	1992–2004	new femoral component + polyethylene tibial insert	65	91	NA		
Hsu et al.	2007	28	1996–2004	static PMMA (n = 7)	101 (63–120)	86	78	81 ^a^	
				articulating PMMA (n = 21)	58 (27–96)	91	95	89 ^a^	
Mittal et al.	2007	37	1987–2003	articulating or static PMMA	51 (24–111)	75	NA		d
Abstracts									
Pietsch et al.	2006	33	2000–2003	RPS	28 (12–48)	91		87 ^b^	e
Souillac et al.	2006	28	2000–2003	articulating PMMA	(20–48)	86	NA		

NA: not available; PMMA: polymethylmethacrylate bone cement; RPS: resterilized prosthesis spacer

A Study

B No. of knees

C Year(s) of collection

D Spacer

E Follow-up, months (range)

F Infection eradication rate, %

G Range of motion, degrees

H Clinical outcome

a Knee Society score

b Hospital for Special Surgery knee score

I Note

c short-term antibiotic therapy

d resistant organisms: methicillin-resistant Staphylococcus aureus/epidermidis

e prospective study

Because of the heterogeneity of the studies and their materials, we could not use meta-analytical techniques. Instead, the studies were reviewed descriptively, which limits the objectivity of conclusions and leaves room for interpretative disagreement. To minimize the confounding effect caused by heterogeneous materials, only papers reporting a pure series of either one-stage or two-stage revision for deep knee infection were included. Consequently, some potentially relevant papers (Rand et al. 1983, Bengtson and Knutson 1991, Hanssen et al. 1994) were excluded. Despite these exclusions, the papers included represent substantial experience in two-stage exchange arthroplasty especially, with over 3,000 person-years of follow-up documented.

There was—and there still is—no question about the role of the two-stage approach being the gold standard in the management of infected knee replacements. Thus, our aim was to detect any factors that might have a relationship with the outcome variables under study. A trend suggesting such a relationship was found between the type of spacer and the rate of recurrence of infections, and also between the type of spacer and postoperative range of motion (or maximal flexion). However, as it appears that recurrence rate has declined over the past 2 decades (Figure 2; Goldman et al. 1996, Hirakawa et al. 1998, Haleem et al. 2004, Sheng et al. 2006), and articulating spacers have been introduced quite recently, it is unclear whether the low recurrence rate with articulating spacers is due to a decline in general recurrence rate or the type of spacer per se. With clinical scores and range of motion, the case-to-case variation was high and definitive conclusions could not be drawn from the present data. The results of the comparative studies (Fehring et al. 2000, Emerson et al. 2002, Jämsen et al. 2006, Hsu et al. 2007) may have been biased due to the use of historical control groups, or to differences in the length of follow-up.

Spacers to be used during the interim period between resection and reimplantation operations were introduced to maintain joint cavity and to prevent contractures of periarticular soft tissues and, to thereby facilitate the reimplantation. Mobile spacers are thought to ease patients’ ambulation and prevent soft tissue contractures and muscle atrophy. In our study, no factors that could be reliably related to improved postoperative outcome were detected. A slight trend suggesting some advantage of mobile spacers over other types was seen, but only concerning range of motion (Figure 3).

Though advantages of spacers of any type could not be proven, the results of our review and of the most recent reports (Hart and Jones 2006, Huang et al. 2006, Jämsen et al. 2006, Hsu et al. 2007) strongly suggest that the use of articulating spacers—or even definitive new knee prostheses (Trezies et al. 2006)—does not affect the chances of eradicating an infection. However, foreign material and even antibiotic-containing bone cement may provide ground for microbial adhesion and bacterial growth (van de Belt et al. 2000, Neut et al. 2005); thus, spacers of any kind may encourage recurrence of infection (Hart and Jones 2006, Jämsen et al. 2006). One should be prepared to remove an already implanted spacer and to redebride the joint in order to achieve control over the infection when markers of inflammation do not normalize or when the symptoms continue after the first-stage operation. For the same reason, two-stage revision (even without a spacer) may be the best approach in complicated cases, since a higher treatment failure rate has been reported in multiply-operated knees (Hart and Jones 2006), in the case of resistant pathogens such as methicillin-resistant staphylococci (Mittal et al. 2007), and in compromised hosts.

A deep infection is an expensive complication (Bengtson et al. 1989, Sculco 1993, 1995, Hebert et al. 1996, Bozic and Ries 2005) and the outcome of two-stage revisions is probably worse than that of aseptic revisions (Barrack et al. 2000a, b, Wang et al. 2004). For these reasons, there has been increased interest in easier ways of managing infected TKAs. Unfortunately, pure antibiotic therapy, lavage procedures, and debridement with retention of the prosthesis have very limited indications (Silva et al. 2002, Leone and Hanssen 2006). Thus, the question in practice is whether one-stage revision is an acceptable approach or not—or how the protocol of two-stage revisions could be improved.

Although reinfection rates of only 9% and 11% after one-stage revision have been reported (Göksan and Freeman 1992, Buechel et al. 2004), the high failure rate of 27% per primum intentionem reported by von Foerster et al. (1991) indicates that the outcome is not as predictable as that of staged exchange. No cost-effectiveness analyses have been published. Considering the probably catastrophic outcome of repeat revisions for septic failure, further studies are required to define the indications for this approach.

Of the different modifications of the conventional two-stage approach, the recently reported protocol with shorter (2–3-week) antibiotic treatment has resulted in reasonable eradication rates (Hoad-Reddick et al. 2005, Hart and Jones 2006), but at present only short-term follow-up is available and there have been no published studies comparing short antibiotic treatment with extensive antibiotic treatment. In most of the studies reviewed, a 4–6-week regimen was used and the antibiotics were selected according to the results of bacterial culture, but few publications have given details of the antibiotic treatment. This can be interpreted as indicating confidence in the importance of surgical intervention as opposed to the use of postoperative antibiotics, although the use of antibiotic-impregnated cement as local antibiotic carrier seems to be widely accepted. The optimal type and length of antibiotic therapy in exchange arthroplasty could not be gleaned from the results of this review.

One of the major problems with the comparisons between the different treatment approaches used in this study is the possibility of selection bias. It is possible that the patients selected for one-stage revision or two-stage revision with mobile spacer prosthesis were healthier than those patients for whom two-stage revision was performed, for example, without any spacers. In such cases, the comparisons may be biased in favor of treatment alternatives, to optimize the changes to eliminate the infection. Such selection by surgeon’s preference would bias comparisons in favor of treatment alternatives other than the conventional two-stage exchange arthroplasty. Inclusion and exclusion criteria, and also the primary diagnoses and patient comorbidity indices were seldom reported. For these reasons, the confounding effects of host status and type of infection—and also of possible selection bias—could not be analyzed.

The studies reviewed report rather similar success rates of 82–100% in eradication of infection. The rates are comparable to those of the more recently published series and to those of series published in languages other than English (Tables 2 and 4). The survival rates (77–80% at 10 years) (Goldman et al. 1996, Hirakawa et al. 1998, Haleem et al. 2004) are similar to the published survival rates of 85–90% following revision arthroplasty for infection in a nationwide series in Finland (Sheng et al. 2006). Thus, it seems that the reports from specialized hospitals do not give over-optimistic results but that similar results can be obtained in different institutions.

The factors that affect the outcome of exchange arthroplasty remain largely unknown. Clinical experience and studies on infected hip and knee arthroplasty suggest that host status (comorbidity), type of infection (acute vs. chronic), and the condition of the environment of the joint involved contribute to the outcome of infected knee replacement (McPherson et al. 1999, 2002). These or any other factors that contribute to the outcome were not adequately analyzed in any of the studies reviewed. Because of the lack of detailed data on treatment failures, such analyses could not be performed using the results of this systematic review either. Future research should focus on the factors affecting the outcome of different treatment approaches.

### Conclusions

Most reports on exchange arthroplasty performed for infected knee arthroplasty are of poor methodological quality, and no unbiased comparative studies exist. The previously reported series included in this systematic analysis yielded similar success rates, but the factors that could predict the outcome are mostly unknown. In the light of our findings, two-stage revision with delayed reimplantation remains the gold standard. It seems reasonable to employ mobile spacers where possible, as they do not compromise the attempts to eradicate infection but may improve postoperative outcome. Despite some promising preliminary reports, the value of and indications for direct exchange arthroplasty remain unclear. Future studies focusing on these treatments should preferably use a prospective randomized setting and compare the new approach to the gold standard of two-stage exchange arthroplasty.

## Appendix 1. Data sources and strategy of the literature review

**Table:**

Literature databases reviewed:
Medline 1966 to present (via Ovid), Medline Daily Update (via Ovid), Medline In-Process and Other Non-indexed Citations (via Ovid), CINAHL (via Ovid), British Nursing Index (via Ovid), Cochrane Central Register of Controlled Trials (via Ovid), Cochrane Database of Systematic Reviews (via Ovid), Database of Abstracts of Reviews of Effects (via Ovid), ACP Journal Club (via Ovid), NHS Health Technology Assessment (via CRD), NHS Economic Evaluation Database (via CRD).
Search strategy for Medline and CINAHL:
1) arthroplasty, replacement, knee/
2) knee prosthesis/
3) “knee prosthes$”.mp
4) “knee arthroplast$”.mp
5) “knee replacement?”.mp
6) reoperation/
7) “revis$”.mp
8) “reimplant$”.mp
9) “exchang$”.mp
10) prosthesis-related infections/
11) infection/
12) wound infection/
13) surgical wound infection/
14) “infect$”.mp
15) (1 or 2 or 3 or 4 or 5) and (6 or 7 or 8 or 9) and (10 or 11 or 12 or 13 or 14)
Search strategy for EBM databases: Cochrane Central Register of Controlled Trials, Database or Abstracts of Reviews of Effects, Cochrane Database of Systematic Reviews, ACP Journal Club (via Ovid):
1) knee replacement?.mp
2) knee arthroplast$.mp
3) knee prosthes$.mp
4) arthroplasty, replacement, knee.mp
5) reoperat$.mp
6) revis$.mp
7) reimplant$.mp
8) exchang$.mp
9) infect$.mp
10) prosthesis-related infection?.mp
11) (1 or 2 or 3 or 4) and (5 or 6 or 7 or 8) and (9 or 10)
Search strategy for Science Citation Index:
1) TS=arthroplasty
2) TS=infection
3) TS=knee
4) TS=(revision OR reoperation OR reimplantation OR exchange)
5) #1 AND #2 AND #3 AND #4
Search strategy for Health Technology Assessment, NHS Economic Evaluation Database:
1) prosthesis-related infections (subject headings)
2) arthroplasty-replacement-knee (subject headings) OR knee-prosthesis (subject headings) AND infect (all fields)
3) knee replacem OR knee arthroplas AND infect

## Appendix 2. The form used for assessment of study quality

**Table:**

STUDY QUALITY Question: Yes; No; Unclear
Was the patient population described in sufficient detail so that you could compare it to the patient population you treat or to the materials of other studies on the same subject?
– Average age
– Male-to-female ratio
– Indications for primary knee replacement
– ASA classification
– Pathogens idenepsied
– Preoperative Knee Society (KSS) or Hospital for Special Surgery (HSS) knee score
– Preoperative range of motion
Was the intervention described in sufficient detail so that you could provide the same treatment for your own patiens?
– Use of antibiotics peroperatively
– The type of spacer used in two-stage revisions
– The length of interim period between the stages in two-stage revisions
Was the associated treatment/rehabilitation described in sufficient detail so that you could provide the same for your own patients?
– Use of antibiotics postoperatively
– Rehabilitation
Were the primary outcome variables reported?
– Number of all post-treatment infections
– Number of reinfections
– Postoperative KSS or HSS
– Postoperative range of motion
Were both advantages and disadvantages of the treatment presented?
Was the proportion of patients excluded less than 20% of the number of patients included?
Was the species of infecting pathogen used as exclusion criterion?
Was loss to follow-up less than 20%?

## References

[CIT0001] Barrack RL, Butler RA, Andrews P, Rorabeck CH, Engh G (2000a). Managing the infected knee: as good as it gets.. Orthopedics.

[CIT0002] Barrack RL, Engh GA, Rorabeck C, Sawhney J, Woolfrey M (2000b). Patient satisfaction and outcome after septic versus aseptic revision total knee arthroplasty.. J Arthroplasty.

[CIT0003] Bengtson S, Knutson K (1991). The infected knee arthroplasty: a 6-year follow-up of 357 cases.. Acta Orthop Scand.

[CIT0004] Bengtson S, Borgquist L, Lindgren L (1989). Cost analysis of prophylaxis with antibiotics to prevent infected knee arthroplasty.. Br Med J.

[CIT0005] Booth RE, Lotke PA (1989). The results of spacer block technique in revision of infected total knee arthroplasty.. Clin Orthop.

[CIT0006] Borden LS, Gearen PF (1987). Infected total knee arthroplasty: a protocol for management.. J Arthroplasty.

[CIT0007] Bozic KJ, Ries MD (2005). The impact of infection after total hip arthroplasty on hospital and surgeon resource utilization.. J Bone Joint Surg (Am).

[CIT0008] Buechel FF, Femino FP, D'alessio J (2004). Primary exchange revision arthroplasty for infected total knee replacement: a long term study.. Am J Orthop.

[CIT0009] Cohen JC, Hozack WJ, Cucker KM, Booth RE (1988). Two-stage reimplantation of septic total knee arthroplasty.. J Arthroplasty.

[CIT0010] Cuckler JM (2005). The infected total knee: management options.. J Arthroplasty (Suppl 2).

[CIT0011] Durbhakula SM, Czajka J, Fuchs MD, Uhl RL (2004). Antibiotic-loaded articulating cement spacer in the 2-stage exchange of infected total knee arthroplasty.. J Arthroplasty.

[CIT0012] Emerson RH, Muncie M, Tarbox TR, Higgins LL (2002). Comparison of a static with a mobile spacer in total knee infection.. Clin Orthop.

[CIT0013] Fehring TK, Odum S, Calton TF, Mason JB (2000). Articulating versus static spacers in revision total knee arthroplasty for sepsis.. Clin Orthop.

[CIT0014] Gacon G, Laurencon M, van de Velde D, Giudicelli DP (1997). Two stages reimplantation for infection after knee arthroplasty. Apropos of a series of 29 cases.. Rev Chir Orthop Reparatrice Appar Mot.

[CIT0015] Göksan SB, Freeman MA (1992). One-stage reimplantation for infected total knee arthroplasty.. J Bone Joint Surg (Br).

[CIT0016] Goldman RT, Scuderi GR, Insall JN (1996). 2-stage reimplantation for infected total knee replacement.. Clin Orthop.

[CIT0017] Gusso MI, Capone A, Civinini R, Scoccianti G (1995). The spacer block technique in revision of total knee arthroplasty with septic loosening.. Chir Organi Mov.

[CIT0018] Haddad FS, Masri BA, Campbell D, Mcgraw RW, Beauchamp CP, Duncan CP (2000). The PROSTALAC functional spacer in two-stage revision for infected knee replacements.. J Bone Joint Surg (Br).

[CIT0019] Haleem AA, Berry DJ, Hanssen AD (2004). Mid-term to long-term followup of two-stage reimplantation for infected total knee arthroplasty.. Clin Orhop.

[CIT0020] Hanssen AD, Rand JA, Osmon DR (1994). Treatment of the infected total knee arthroplasty with insertion of another prosthesis.. Clin Orthop.

[CIT0021] Hart WJ, Jones RS (2006). Two-stage revision of infected total knee replacements using articulating cement spacers and short-term antibiotic therapy.. J Bone Joint Surg (Br).

[CIT0022] Hebert CK, Williams RE, Levy RS, Barrack RL (1996). Cost of treating an infected total knee replacement.. Clin Orthop.

[CIT0023] Henderson MH, Booth RE (1991). The use of an antibiotic-impregnated spacer block for revision of the septic total knee arthroplasty.. Semin Arthroplasty.

[CIT0024] Hirakawa K, Stulberg BN, Wilde AH, Bauer TW, Secic M (1998). Results of 2-stage reimplantation for infected total knee arthroplasty.. J Arthroplasty.

[CIT0025] Hoad-reddick DA, Evans CR, Norman P, Stockley I (2005). Is there role for extended antibiotic therapy in a two-stage revision of the infected knee arthroplasty.. J Bone Joint Surg (Br).

[CIT0026] Hofmann AA, Kane KR, Tkach TK, Plaster RL, Camargo MP (1995). Treatment of infected total knee arthroplasty using an articulating spacer.. Clin Orhop.

[CIT0027] Hofmann AA, Goldberg T, Tanner AM, Kurtin SM (2005). Treatment of infected total knee arthroplasty using an articulating spacer: 2- to 12-year experience.. Clin Orthop.

[CIT0028] Hsu YC, Cheng HC, Ng TP, Chiu KY (2007). Antibiotic-loaded cement articulating spacer for 2-stage reimplantation in infected total knee arthroplasty: a simple and economic method.. J Arthroplasty.

[CIT0029] Huang HT, Su JY, Chen SK (2006). The results of articulating spacer technique for infected total knee arthroplasty.. J Arthroplasty.

[CIT0030] Insall JN, Thompson FM, Brause BD (1982). Two-stage reimplantation for the salvage of infected total knee arthroplasty.. Orthop Trans.

[CIT0031] Insall JN, Thompson FM, Brause BD (1983). Two-stage reimplantation for the salvage of infected total knee arthroplasty.. J Bone Joint Surg (Am).

[CIT0032] Jämsen E, Sheng P, Halonen P, Lehto MU, Moilanen T, Pajamäki J, Puolakka T, Konttinen YT (2006). Spacer prostheses in two-stage revision of infected knee arthroplasty.. Int Orthop.

[CIT0033] Jhao C, Jiang CC (2003). Two-stage reimplantation without cement spacer for septic total knee replacement.. J Formos Med Assoc.

[CIT0034] Kirschner S, Werner A, Walther M, Rader CP, Gohlke FE (2000). Versorgung mit anatomischen Spacern bei chronischem endoprotheseinfekt am Knie.. Orthop Praxis.

[CIT0035] Lecuire F, Rubini J, Basso M, Benareau I (1999). Traction-mobilization in 2-stage treatment of infected total knee prosthesis. Apropos of 12 cases.. Rev Chir Orthop Reparatrice Appar Mot.

[CIT0036] Leone JM, Hanssen AD (2006). Management of infection at the site of a total knee arthroplasty.. AAOS Instr Course Lect.

[CIT0037] Lonner JH, Beck TD, Rees H, Roullet M, Lotke PA (2001). Results of two-stage revision of the infected total knee arthroplasty.. Am J Knee Surg.

[CIT0038] Lu H, Kou B, Lin J (1997). One-stage reimplantation for the salvage of total knee arthroplasty complicated by infection.. Chin J Surg.

[CIT0039] MacAvoy MC, Ries MD (2005). The ball and socket articulating spacer for infected total knee arthroplasty.. J Arthroplasty.

[CIT0040] Malmivaara A, Koes BW, Bouter LM, van Tulder MW (2006). Applicability and clinical relevance of results in randomized controlled trials: the Cochrane review on exercise therapy for low back pain as an example.. Spine.

[CIT0041] Masri BA, Kendall RW, Duncan CP, Beauchamp CP, Mcgraw RW, Bora B (1994). Two-stage exchange arthroplasty using a functional antibiotic-loaded spacer in the treatment of the infected knee replacement: the Vancouver experience.. Semin Arthroplasty.

[CIT0042] McPherson EJ, Patzakis MJ, Gross JE, Holtom PD, Song M, Dorr LD (1997). Infected total knee arthroplasty. Two-stage reimplantation with a gastrocnemius rotational flap.. Clin Orthop.

[CIT0043] McPherson EJ, Tontz W, Patzakis M, Woodsome C, Holtom P, Norris L, Shufelt C (1999). Outcome of infected total knee utilizing a staging system for prosthetic joint infection.. Am J Orthop.

[CIT0044] McPherson EJ, Woodson C, Holtom M, Roidis N, Shufelt C, Patzakis M (2002). Periprosthetic total hip infection: outcomes using a staging system.. Clin Orthop.

[CIT0045] Meek RM, Masri BA, Dunlop D, Garbuz DS, Greidanus NV, Mcgraw R, Duncan CP (2003). Patient satisfaction and functional status after treatment of infection at the site of total knee arthroplasty with use of the prostalac articulating spacer.. J Bone Joint Surg (Am).

[CIT0046] Meek RM, Dunlop D, Garbuz DS, Mcgraw R, Greidanus NV, Masri BA (2004). Patient satisfaction and functional status after aseptic versus septic revision total knee arthroplasty using the prostalac articulating spacer.. J Arthroplasty.

[CIT0047] Mittal Y, Fehring TK, Hanssen A, Marculescu C, Odum SM, Osmon D (2007). Two-stage reimplantation for periprosthetic knee infection involving resistant organisms.. J Bone Joint Surg (Am).

[CIT0048] Mont MA, Waldman BJ, Hungerford DS (2000). Evaluation of preoperative cultures before second-stage reimplantation of a total knee prosthesis complicated by infection. A comparison-group study.. J Bone Joint Surg (Am).

[CIT0049] Neut D, Hendriks JG, van Horn JR, van der Mei HC, Busscher HJ (2005). Pseudomonas aerigunosa biofilm formation and slime excretion on antibioticloaded bone cement.. Acta Orthop.

[CIT0050] Pietsch M, Wenisch C, Traussnig S, Trnoska R, Hofmann S (2003). Temporary articulating spacer with antibiotic-impregnated cement for an infected knee endoprosthesis.. Orthopade.

[CIT0051] Pietsch M, Hofmann S, Wenisch C (2006). Treatment of deep infection of total knee arthroplasty using a two-stage procedure.. Operative Orthopadie und Traumatologie.

[CIT0052] Pitto RP, Castelli CC, Ferrari R, Munro J (2005). Pre-formed articulating knee spacer in two-stage revision for the infected total knee arthroplasty.. Int Orthop.

[CIT0053] Rand JA, Bryan RS (1983). Reimplantation for the salvage of an infected total knee arthroplasty.. J Bone Joint Surg (Am).

[CIT0054] Rosenberg AG, Haas B, Barden R, Marquez D, Landon GC, Galante JO (1988). Salvage of infected total knee arthroplasty.. Clin Orthop.

[CIT0055] Sculco TP (1993). The economic impact of infected total joint arthroplasty.. AAOS Instr Course Lect.

[CIT0056] Sculco TP (1995). The economic impact of infected joint arthroplasty.. Orthopedics.

[CIT0057] Sheng PY, Konttinen L, Lehto M, Ogino D, Jamsen E, Nevalainen J, Pajamaki J, Halonen P, Konttinen YT (2006). Revision total knee arthroplasty: 1990 through 2002. A review of the Finnish arthroplasty registry.. J Bone Joint Surg (Am).

[CIT0058] Siebel T, Kelm J, Porsch M, Regitz T, Neumann WH (2002). Two-stage exchange of infected knee arthroplasty with a prosthesis-like interim cement spacer.. Acta Orthop Belg.

[CIT0059] Silva M, Tharani R, Schmalzried TP (2002). Results of direct exchange or debridement of the infected total knee arthroplasty.. Clin Orhop.

[CIT0060] Souillac V, Costes S, Aunoble S, Langlois V, Dutronc H, Chauveaux D (2006). Evaluation of an articulated spacer for two-stage reimplantation for infected total knee arthroplasty: 28 cases.. Rev Chir Orthop Reparatrice Appar Mot.

[CIT0061] Trezies A, Parish E, Dixon P, Cross M (2006). The use of an articulating spacer in the management of infected total knee arthroplasties.. J Arthroplasty.

[CIT0062] van de Belt H, Neut D, Schenk W, van Horn JR, van der Mei HC, Busscher HJ (2000). Gentamicin release from polymethylmethacrylate bone cements and Staphylococcus aureus biofilm formation.. Acta Orthop Scand.

[CIT0063] van Tulder MW, Assendelft WJ, Koes BW, Bouter LM (1997). Method guidelines for systematic reviews in the Cochrane Collaboration Back Review Group for spinal disorders.. Spine.

[CIT0064] von Foerster G, Kluber D, Kabler U (1991). Mid- to long-term results after treatment of 118 cases of periprosthetic infections after knee joint replacement using one-stage exchange surgery.. Orthopade.

[CIT0065] Wang CJ, Hsieh MC, Huang TW, Wang JW, Chen LS, Liu CY (2004). Clinical outcome and patient satisfaction in aseptic and septic revision total knee arthroplasty.. Knee.

[CIT0066] Whiteside LA (1994). Treatment of infected total knee arthroplasty.. Clin Ortho.

[CIT0067] Wilde AH, Ruth JT (1988). Two-stage reimplantation in infected total knee arthroplasty.. Clin Orthop.

[CIT0068] Windsor RE, Insall JN, Urs WK, Miller DV, Brause BD (1990). Two-stage reimplantation for the salvage of total knee arthroplasty complicated by infection. Further follow-up and refinement of indications.. J Bone Joint Surg (Am).

